# Protein-protein interaction network and mechanism analysis in ischemic stroke

**DOI:** 10.3892/mmr.2014.2696

**Published:** 2014-10-17

**Authors:** ZHE QUAN, YUAN QUAN, BO WEI, DENING FANG, WEIDONG YU, HAO JIA, WEI QUAN, YUGUANG LIU, QIHONG WANG

**Affiliations:** 1 Department of Neurosurgery, Qilu Hospital, Shandong University, Jinan, Shandong 250000, P.R. China; 2 Department of Neurosurgery, Shanghai Fengxian District Central Hospital, Shanghai 201400, P.R. China; 3 Norman Bethune Medical School of Jilin University, Changchun, Jilin 130000, P.R. China; 4 Department of Neurosurgery, The Third Affiliated Hospital (China-Japan Union Hospital) of Jilin University, Changchun, Jilin 130031, P.R. China; 5 Department of Gastroenterology, Shanghai No. 8 Hospital, Shanghai 200235, P.R. China; 6 Department of Emergency Medicine, The Fourth Affiliated Hospital of China Medical University, Shenyang, Liaoning 110000, P.R. China; 7 Department of Infection, HuiNanXian Hospital, Huinan, Jilin 135100, P.R. China; 8 Department of Neurosurgery, Ruijin Hospital Affiliated to Shanghai Jiaotong University School of Medicine, Shanghai 200025, P.R. China

**Keywords:** ischemic stroke, protein-protein interaction network, differentially expressed gene, middle cerebral artery occlusion

## Abstract

Ischemic stroke is a leading cause of mortality and permanent disability, with enormous financial repercussions on health systems worldwide. Ischemic brain injury results from a complex sequence of pathophysiological events that evolve over time. In order to examine the molecular mechanisms underlying middle cerebral artery occlusion (MCAO)-induced ischemic stroke, the GSE35338 affymetrix microarray data was obtained from the Gene Expression Omnibus database and the differentially expressed genes (DEGs) between samples from patients with MCAO-induced ischemic stroke and sham controls at various time points were identified. Furthermore, protein-protein interaction (PPI) networks were constructed by mapping the DEGs into PPI data to identify the pathways that these DEGS are involved in. The results revealed that the expression of 438 DEGs, which are mainly involved in cell death, oxidant reduction, cell cycle and cell-cell signaling, were altered in MCAO samples. The nodes of CXC motif chemokine 10 (CXCL10) and interleukin-6 (IL-6) were large, with degrees of >20. In conclusion, the results suggest that CXCL10 and IL-6 have important roles in the occurrence and progression of MCAO-induced ischemic stroke.

## Introduction

Ischemic stroke is one of the leading causes of mortality (1) and long-term disability in adults worldwide (2). Three months following a stroke, ~15–30% of stroke survivors are permanently disabled and 20% require costly long-term care (3). Deficits include partial paralysis, and difficulties with memory, thinking, language and movement. According to the current data, ~80% of strokes are ischemic (4). Ischemic strokes result from a transient or permanent reduction in cerebral blood flow that is restricted to the territory of a major brain artery (5). The reduction in flow is, in the majority of cases, caused by middle cerebral artery occlusion (MCAO) either by an embolus or local thrombosis. In the center of the ischemic territory, oxygen and glucose deprivation, neuronal depolarization and Ca^2+^-mediated excitotoxicity induces necrotic and apoptotic cell death (6). The amount of excitotoxicity and oxidative damage in cerebral tissue depends on several factors, including the degree and the duration of ischemia, and the capability of the brain to recover and repair itself (3).

Rigorous laboratory investigations of cerebral ischemia conducted over the past two decades have identified various factors that are involved in the pathogenesis of ischemic stroke, including inflammation, excitotoxicity and ionic imbalance, oxidative and nitrosative stress, as well as apoptotic-like cell death (7). In particular, increasing evidence demonstrates that serological markers of inflammation, including C-reactive protein and soluble intercellular adhesion molecule account for the pathogenic progression of ischemic stroke (8). Despite advances in the understanding of the pathophysiology of ischemic stroke, the precise molecular mechanisms involved in ischemic stroke induced by MCAO remain poorly understood.

Therefore, in the present study, microarrays were utilized to identify the differentially expressed genes (DEGs) between sham samples and MCAO-induced focal ischemic samples at various time-points (1, 3 and 7 days). Gene Ontology (GO)enrichment analysis was performed and a protein-protein interaction (PPI) network was constructed by mapping the DEGs to the PPI data. This information may facilitate the understanding of the molecular mechanisms underlying ischemic stroke and thus aid in selecting an appropriate and effective treatment strategy for patients.

## Materials and methods

### Affymetrix microarray data

The transcriptional profile of GSE35338 (9) was obtained from National Center of Biotechnology Information Gene Expression Omnibus (GEO) database (http://www.ncbi.nlm.nih.gov/geo/), which is based on the Affymetrix Mouse Genome 430 2.0 Array (Affymetrix, Inc., Santa Clara, CA, USA). In total, 21 specimens, obtained one day (n=5), three days (n=3) and seven days (n=3) following MCAO-induced ischemic stroke, and one day (n=4), three days (n=3) and seven days (n=3) following control sham surgery, were available based on the GPL1261 Platform.

### Data preprocessing

The probe-level data in CEL files (Affymetrix Inc.) were converted into expression measures and background correction was performed by the robust multiarray average algorithm (10) with defaulted parameters in the R affy package (11,12). If there were multiple probe sets that corresponded to the same gene, the expression values of those probe sets were averaged.

### DEG analysis

For the GSE 35338 dataset, LIMMA package (13) in R language (Affymetrix Inc.) was used to identify DEGs between the MCAO and sham control samples. Only the DEGs with a fold change value >1.5 and a P-value <0.05 were selected.

### GO and Kyoto Encyclopedia of Genes and Genomes (KEGG) pathway analysis

GO analysis has become a commonly utilized approach for functional annotation of large-scale genomic data (14).

The KEGG pathway database (15) (http://www.genome.jp/kegg/pathway.html) contains information of the manner in which molecules or genes are networked. It is complementary to the majority of the existing molecular biology databases that contain information of individual molecules or individual genes.

The database for annotation, visualization and integrated discovery (DAVID Bioinformatics Resources 6.7; http://david.abcc.ncifcrf.gov/home.jsp), a high-throughput and integrated data-mining environment, analyzes gene lists derived from high-throughput genomic experiments (16). In the present study, DAVID was used to identify over-represented GO categories in biological processes and significant pathways with a value of P<0.05.

### PPI network construction

To demonstrate the potential PPI correlation, the DEGs were mapped to the PPI data that were collected from the Search Tool for the Retrieval of Interacting Genes (STRING) (17) database. STRING is a large dataset containing functional links between proteins on the basis of experimental evidence for PPIs as well as interactions predicted by comparative genomics and text mining. It uses a scoring system that is intended to reflect the evidence of predicted interactions. In the present study, interactions with a score ≥0.7 were included. Next, a PPI network was constructed by Cytoscape (18) based on the PPI correlations.

### Molecular Complex Detection (MCODE) analysis

MCODE (ftp://ftp.mshri.on.ca/pub/BIND/Tools/MCODE) detects densely connected regions in large PPI networks that may represent molecular complexes (19). In the present study, clusters of highly intra-connected nodes (n>10) in the network were searched using an MCODE plug-in in the Cytoscape network. Next, the identified clusters were used for functional enrichment analysis.

## Results

### DEG selection

**Figure 1:**
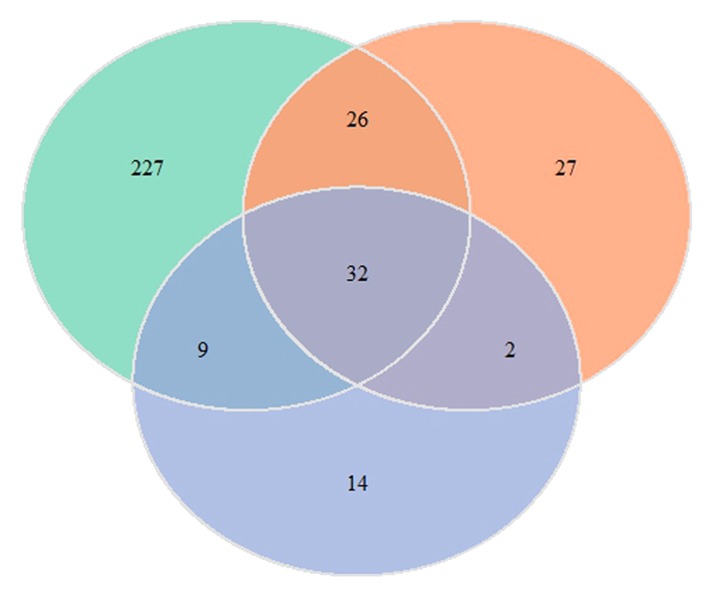
Venn diagram demonstrating that at one (green), three (orange) and seven (blue) days following MCAO, the samples have overlapping but distinct sets of DEGs. A total of 337 DEGs are identified in the MCAO samples, including 227 distinct DEGs in samples obtained one day following MCAO, 27 distinct DEGs in samples obtained three days following MCAO and 14 distinct DEGs obtained seven days following MCAO. DEGs, differentially expressed genes; MCAO, middle cerebral artery occlusion.

In order to obtain the DEGs between MCAO reactive astrocytes and sham controls at various time points, publically available microarray datasets were obtained from the GEO. A total of 294 genes were selected as DEGs between samples obtained from one day following MCAO and sham specimens; 87 DEGs between samples from three days following MCAO and sham samples; and 57 DEGs between samples from seven days following MCAO and sham controls with a fold-change >1.5 and P<0.05. The samples obtained from one, three and seven days following MCAO had overlapping but distinct sets of DEGs. The Venn diagram (Fig. 1) demonstrates that 32 genes are common to the three MCAO samples and all of these genes were upregulated in the MCAO-reactive astrocytes. There were 227, 27 and 14 distinct DEGs in the samples taken from one, three and seven days following MCAO, respectively.

### GO enrichment analysis of DEGs

To investigate the functional changes in the pathological course of MCAO, the DEGs were mapped to the GO database. This project provided three structured networks of defined terms to describe the gene product attributes: Biological process (BP), molecular function (MF) and cellular compartment (CC). In the present study, the majority of the enriched genes were upregulated in the MCAO samples, particularly in the samples from seven days following MCAO. The DEGs for the samples taken from one day following MCAO were most commonly associated with BP and CC, including the extracellular region, response to wounding and immune response (Table I). Similarly, the DEGs in samples taken from three days following MCAO were also mainly associated with BP and CC, for instance, extracellular region, cell cycle, response to wounding and defense response (Table II). Table III demonstrates that the enriched GO terms of DEGs in the samples taken from seven days following MCAO were correlated with all of the three defined terms. The enriched BP GO terms included immune response, response to wounding and inflammatory response. The enriched CC GO terms included extracellular region and extracellular space. The enriched MF GO terms included Ca^2+^ ion binding and enzyme inhibitor activity. In addition, significantly enriched GO terms with high counts of distinct DEGs in each MCAO sample were identified. The enriched terms of the distinct DEGs in samples obtained from one day following MCAO included cell death, oxidation reduction and response to wounding (Table V). The enriched terms of the distinct DEGs in samples obtained from three days following MCAO included cell cycle, cell division and nuclear division. The enriched term of the specific DEGs in samples obtained from seven days following MCAO was cell-cell signaling.

### Pathway enrichment analysis

To gain further insights into the changes in the biological pathways in the cells in the MCAO samples, the online biological classification tool DAVID was used and significant enrichment of these DEGs in multiple KEGG terms was observed (Table IV). The most significantly enriched pathway that the DEGs in samples from one day following MCAO were involved in was cytokine-cytokine receptor interaction. The most significantly enriched pathway that the DEGs in samples from three days following MCAO were involved in was the p53 signaling pathway. The DEGs in this group were also shown to be involved in the cell cycle, cytokine-cytokine receptor interaction and cytosolic DNA-sensing pathway. In the samples from seven days following MCAO, the pathways correlated with the DEGs were cytokine-cytokine receptor interaction, the nucleotide-binding oligomerization domain (NOD)-like receptor signaling pathway and the chemokine signaling pathway.

### PPI network construction

**Figure 2:**
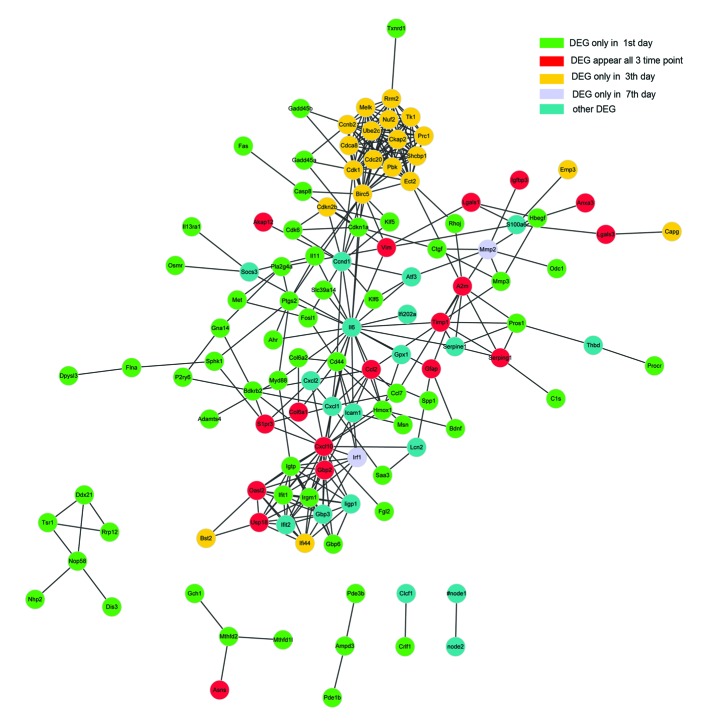
PPI network constructed in MCAO-induced sample at various time points. PPI, protein-protein interactions; MCAO, middle cerebral artery occlusion.

**Figure 3:**
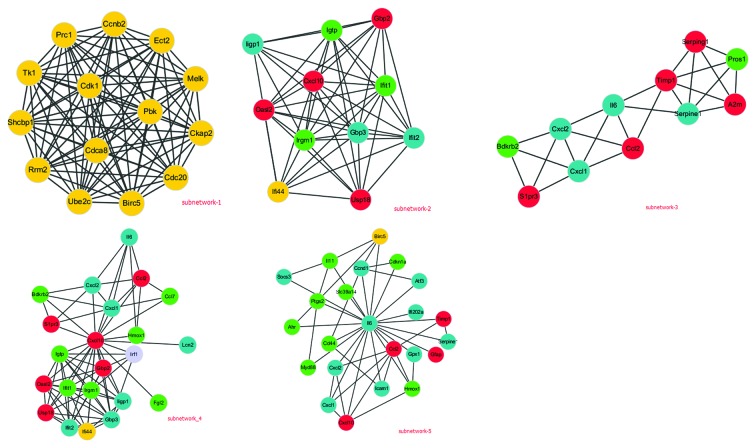
Subnetwork clusters identified from the PPI network. The red nodes indicates the DEGs that were common to all MCAO samples. The green nodes indicate distinct DEGs in the samples obtained one day following MCAO. The orange nodes represent distinct DEGs in the three days following MCAO samples. The purple nodes represent the distinct DEGs the seven days following MCAO samples. PPI, protein-protein interactions; DEGs, differentially expressed genes; MCAO, middle cerebral artery occlusion.

To construct the PPI network, PPI data was obtained from the STRING database. In the network, each edge is examined by a score as the edge weight to quantify the interaction confidence. To obtain the correlations, the PPIs with a score of ≥0.7 were selected (Fig. 2). Next, the degree of each node in the networks was calculated by iGrph, a publicly available R package for analyzing graphs. The degree is the number of edges connecting all of the nodes in the network. A higher value for the degree indicates a highly connected network and is likely to be more robust. A total of 22 nodes were screened with degrees >10. Notably, the degrees of CXC motif chemokine 10 (CXCL10) and interleukin-6 (IL-6) were >20, suggesting they may have an important role in MCAO-induced ischemia. In Fig. 3, these two DEGs as well as their first nodes formed local networks (sub-network 4 and sub-network 5). In addition, the network was further analyzed by MCODE and three sub-networks (sub-network 1–3) were searched with the intra-connected nodes >10. The functions of these sub-networks were mainly correlated with the cell cycle, immune response, response to wounding and regulation of cell proliferation.

## Discussion

Stroke is one of the most common causes of mortality and disability, with marked financial repercussions on health systems worldwide (20). Altered gene expression is an important feature of ischemic cerebral injury and affects proteins in numerous functional classes (21). Therefore, an understanding of the molecular mechanisms underlying this disease is critically important for developing effective management strategies. In the present study, a bioinformatics method was utilized to examine the molecular mechanism of MCAO-induced ischemic stroke development at various time points. A total of 337 DEGs were identified between the MCAO and sham control samples. These genes included 227 distinct DEGs in the samples obtained from one day following MCAO, 27 distinct DEGs in the samples from three days following MCAO and 14 distinct DEGs in the samples from seven days following MCAO. The cytokine-cytokine receptor interaction pathway, p53 signaling pathway and mitogen-activated protein kinase (MAPK) signaling pathway were dysregulated in the MCAO samples. By mapping DEGs to a PPT database, a PPT network was constructed, which revealed the interaction of DEGs. Through this network, it was identified that the node magnitude of CXCL10 and IL-6 were larger with degrees of >20.

CXCL10, a chemokine that targets activated T cells and natural killer cells expressing CXCR3, has been implicated in inflammatory disease and is most commonly associated with T cell responses (22–25). CXCL10 is expressed by neurons in response to brain injury and leads to the recruitment of microglia for the purpose of dendritic reorganization (26). Exogenous application of CXCL10 has been demonstrated to induce neuronal apoptosis and to inhibit herpes simplex virus replication in neurons *in vitro* (27). The CXCL10 chemokines appear to be essential for immune cell activation and trafficking of peripheral immune cells across the blood-brain barrier (28,29). Previously, CXCL10 has also been reported to have an important role in ischemia/reperfusion-induced liver inflammation and hepatocellular injury (25). In the present study, CXCL10 acted as a hub node in the network suggesting this gene has an important role in ischemic stroke development and may be used as a specific therapeutic molecular target in the treatment of ischemic stroke.

IL-6 is an acute phase reactant cytokine with pro- and anti-inflammatory properties (30). IL-6 is produced by several cell types, including fibroblasts, monocytes, adipocytes and endothelial cells (31). IL-6 has been demonstrated to be able to modulate cardiovascular function and exert a negative inotropic effect via nitric oxide-dependent pathways (32,33). An increasing number of experimental observations suggest that IL-6 has a central role in the pathogenesis of several ischemic cardiovascular disorders, including unstable angina (34) and acute coronary syndromes (35). Furthermore, IL-6 is also considered to be associated with the initiation of liver regeneration in mice (30). In humans, IL-6 is involved in the acute phase response that follows cerebral ischemia, and there is a correlation between high plasma levels of IL-6 and occurrence of early neurological deterioration following stroke (36) and progression of lacunar infarction (37). In accordance with the present findings, Flex *et al* (38) also suggested that IL-6 is significantly and independently associated with a history of ischemic stroke.

From the results of GO enrichment analysis, it was identified that the majority of enriched GO terms of DEGs in the samples obtained from one day following MCAO were correlated with cell death and oxidant reduction. This suggested that cell death and the lack of oxygen may have an important role in the onset of MCAO-induced ischemic stroke. This finding is consistent with that of a study by Mergenthaler *et al* (39), which suggested that programmed cell death was initiated hours following ischemia onset and lasted over a number of days. Oxidative stress contributes to the pathogenesis of a number of neurological conditions, including stroke. Its involvement in ischemic cell death results from the formation of ROS/reactive nitrogen species through multiple injury mechanisms (3). By three and seven days following MCAO, the majority of the DEGs enriched in GO terms were associated with the cell cycle and cell-cell signaling, respectively. This indicated that cell proliferation and cell-cell signaling may be essential in the pathogenesis of ischemic stroke development. These results are consistent with a previous study by Zamanian *et al* (9) who reported that the expression of numerous genes associated with the cell-cycle, including late-phase cyclin B and cyclin-dependent kinase Cdk1, were not induced one day following MCAO but were elevated 3-fold to 4-fold in MCAO reactive astrocytes three days later. The results of GO enrichment analysis also indicated that ischemic brain injury results from a complex sequence of pathophysiological events that evolve over time.

The resulting PPI network is unweighted, since each PPI occurred only once. As it is too large to yield more specific information, it is necessary to divide the network into sub-networks, which may represent functional modules or protein sub-complexes. In the present study, clustering using MCODE and first hub nodes identified five sub-networks. The main functions of subnetwork-2 and subnetwork-4 were correlated with the immune response. Lakhan *et al* (3) reported that severe brain ischemia perturbed innate and adaptive immune cells, resulting in systemic immunodepression that predisposes stroke patients to life-threatening infections. Manipulation of the immune system through mucosal tolerance may provide a novel tool for stroke prophylaxis in humans (7). Notably, all of the DEGs enriched in subnetwork-1 were only observed in the samples obtained from three days following MCAO, whose GO terms were cell cycle and cell division, suggesting that they may be involved in the processes of the cell cycle.

In conclusion, the present study analyzed the gene expression profiles and pathways that may be involved in the progression of MCAO-induced ischemic stroke by using comprehensive bioinformatics analysis. It was identified that CXCL10 and IL-6 may have important roles in the progression of ischemic stroke and thus may be used as specific therapeutic molecular targets. Furthermore, ischemic brain injury resulted from a complex sequence of pathophysiological events that evolved over time. Notably, cell-cycle genes were only induced three days following MCAO. However, further studies are required to confirm these observations and determine their clinical utility in the therapeutic management of ischemic stroke.

## Figures and Tables

**Table I:** Top ten significantly enriched GO terms with a high count of DEGs in the samples one day following MCAO.

Term	Category	Description	Count	P-value
GO: 0005886	CC	Plasma membrane	50	2.34E-02
GO: 0005576	CC	Extracellular region	49	4.91E-08
GO: 0044421	CC	Extracellular region part	35	2.22E-10
GO: 0009611	BP	Response to wounding	27	6.07E-13
GO: 0006955	BP	Immune response	23	2.53E-07
GO: 0005615	CC	Extracellular space	23	7.05E-07
GO: 0042127	BP	Regulation of cell proliferation	22	8.30E-06
GO: 0006952	BP	Defense response	21	1.86E-06
GO: 0008219	BP	Cell death	20	4.01E-05
GO: 0016265	BP	Death	20	5.50E-05

GO, Gene Ontology; DEGs, differentially expressed genes; MCAO, middle cerebral artery occlusion; CC, cellular compartment; BP, biological process.

**Table II:** Top ten significantly enriched GO terms with a high count of DEGs in the samples three days following MCAO.

Term	Category	Description	Count	P-value
GO: 0005576	CC	Extracellular region	18	2.24E-04
GO: 0044421	CC	Extracellular region part	13	4.90E-05
GO: 0007049	BP	Cell cycle	12	1.36E-04
GO: 0042127	BP	Regulation of cell proliferation	11	2.19E-04
GO: 0009611	BP	Response to wounding	10	3.77E-05
GO: 0051301	BP	Cell division	9	5.50E-05
GO: 0006955	BP	Immune response	9	1.74E-03
GO: 0005615	CC	Extracellular space	9	9.95E-04
GO: 0000278	BP	Mitotic cell cycle	8	1.58E-04
GO: 0022403	BP	Cell cycle phase	8	9.39E-04

GO, Gene Ontology; DEGs, differentially expressed genes; MCAO, middle cerebral artery occlusion; CC, cellular compartment; BP, biological process.

**Table III:** Top ten significantly enriched GO terms with a high count of DEGs in the samples seven days following MCAO.

Term	Category	Description	Count	P-value
GO: 0005576	CC	Extracellular region	21	3.08E-08
GO: 0044421	CC	Extracellular region part	16	6.15E-09
GO: 0005615	CC	Extracellular space	13	3.75E-08
GO: 0006955	BP	Immune response	11	1.56E-06
GO: 0009611	BP	Response to wounding	9	1.11E-05
GO: 0006952	BP	Defense response	9	6.89E-05
GO: 0006954	BP	Inflammatory response	8	6.03E-06
GO: 0005509	MF	Calcium ion binding	8	1.34E-02
GO: 0042127	BP	Regulation of cell proliferation	7	6.57E-03
GO: 0004857	MF	Enzyme inhibitor activity	6	8.59E-04

GO, Gene Ontology; DEGs, differentially expressed genes; MCAO, middle cerebral artery occlusion; CC, cellular compartment; BP, biological process; MF, molecular function.

**Table IV:** Enriched KEGG pathway of DEGs in MCAO samples one day following MCAO.

Pathway	Genes	P-value
One day following MCAO		
Cytokine-cytokine receptor interaction	CXCL1, IL-6, CCL2, CCL7, OSMR, MET, CXCL2, EDA2R, CXCL10, IL11, CLCF1, FAS, IL13RA1, TNFRSF12A	5.87E-05
p53 signaling pathway	CDKN1A, CCND1, SERPINE1, CASP8, RPRM, CDK6, FAS, GADD45B, IGFBP3, GADD45A	6.80E-07
MAPK signaling pathway	MAP3K6, PLA2G4A, BDNF, HSPB1, FAS, GADD45B, FLNC, GADD45A, CD14, FLNA	1.706E-02
Focal adhesion	CCND1, CAV1, MET, COL6A2, COL6A1, FLNC, FLNA, SPP1	2.83E-02
Complement and coagulation cascades	A2M, THBD, SERPINE1, SERPING1, C1S, BDKRB2, PROS1	8.37E-04
Jak-STAT signaling pathway	CCND1, IL-6, CLCF1, OSMR, SOCS3, IL13RA1, IL11	2.59E-02
NOD-like receptor signaling pathway	CXCL1, IL-6, CCL2, CXCL2, CASP8, CCL7	2.23E-03
Toll-like receptor signaling pathway	IL-6, MYD88, CASP8, CD14, CXCL10, SPP1	1.60E-02
Hematopoietic cell lineage	IL-6, CD44, CD24A, CD14, IL-11	3.67E-02
Melanoma	CDKN1A, CCND1, MET, CDK6	8.99E 02
Three days following MCAO		
p53 signaling pathway	CDK1, CCND1, CCNB2, RRM2, SERPINE1, IGFBP3	2.45E-05
Cell cycle	CDK1, CCND1, CCNB2, CDKN2B, CDC20	4.13E-03
Cytokine-cytokine receptor interaction	IL-6, CCL2, CLCF1, TNFRSF12A, CXCL10	3.67E-02
Cytosolic DNA-sensing pathway	IFI202B, IL6, CXCL10	3.31E-02
Seven days following MCAO		
Cytokine-cytokine receptor interaction	CXCL1, CCL2, CXCL2, TNFSF9, CXCL10	7.46E-03
NOD-like receptor signaling pathway	CXCL1, CCL2, CXCL2, TNFAIP3	1.03E-03
Chemokine signaling pathway	CXCL1, CCL2, CXCL2, CXCL10	2.09E-02

KEGG, Kyoto Encyclopedia of Genes and Genomes; DEGs, differentially expressed genes; MCAO, middle cerebral artery occlusion; MAPK, mitogen-activated protein kinase; Jak-STAT, janus kinase-signal transducers and activators of transcription; NOD, nucleotide-binding oligomerization domain; IL-6, interleukin-6; CXCL10, CXC motif chemokine 10.

**Table V:** Significantly enriched GO terms with a high count of distinct DEGs in MCAO samples.

Term	Description	Count	P-value
One day after MCAO			
GO: 0008219	Cell death	16	1.32E-04
GO: 0016265	Death	16	5.22E-04
GO: 0055114	Oxidation reduction	16	6.65E-04
GO: 0012501	Programmed cell death	15	8.01E-04
GO: 0009611	Response to wounding	14	1.55E-03
GO: 0006915	Apoptosis	14	2.05E-03
GO: 0042127	Regulation of cell proliferation	13	2.82E-03
GO: 0042981	Regulation of apoptosis	12	3.28E-03
GO: 0043067	Regulation of programmed cell death	12	3.67E-03
GO: 0010941	Regulation of cell death	12	7.83E-03
Three days after MCAO			
GO: 0007049	Cell cycle	10	7.63E-08
GO: 0051301	Cell division	8	9.24E-08
GO: 0000280	Nuclear division	7	2.27E-07
GO: 0007067	Mitosis	7	2.27E-07
GO: 0000087	M phase of mitotic cell cycle	7	2.57E-07
GO: :0048285	Organelle fission	7	2.82E-07
GO: 0000278	Mitotic cell cycle	7	9.90E-07
GO: 0000279	M phase	7	2.35E-06
GO: 0022403	Cell cycle phase	7	5.50E-06
GO: 0022402	Cell cycle process	7	1.55E-05
Seven days after MCAO			
GO: 0007267	Cell-cell signaling	3	0.046035188

GO, Gene Ontology; DEGs, differentially expressed genes; MCAO, middle cerebral artery occlusion; BP, biological process; MF, molecular function; CC, cellular compartment.
